# Inhibitory Activity of LDT10 and LDT119, New Saturated Cardanols, Against *Trypanosoma cruzi*

**DOI:** 10.3390/ph19010030

**Published:** 2025-12-22

**Authors:** Renato Granado, Brenda de Lucena Costa, Cleonice Andrade Holanda, Daniel Carneiro Moreira, Luiz Antonio Soares Romeiro, Emile Santos Barrias, Wanderley de Souza

**Affiliations:** 1 Laboratório de Ultraestrutura Celular Hertha Meyer, Instituto de Biofísica Carlos Chagas Filho, Universidade Federal do Rio de Janeiro, Centro de Ciências da Saúde, Bloco G, Ilha do Fundão, Rio de Janeiro 21941-900, RJ, Brazil; 2 Diretoria de Metrologia Aplicada às Ciências da Vida, Instituto Nacional de Metrologia, Qualidade e Tecnologia—Inmetro, Rio de Janeiro 25250-020, RJ, Brazil; 3 Laboratório de Desenvolvimento de Inovações Terapêuticas, Núcleo de Medicina Tropical, Faculdade de Medicina, Universidade de Brasília, Brasília 70910-900, DF, Brazil; 4 Núcleo de Pesquisa em Morfologia e Imunologia Aplicada (NuPMIA), Faculdade de Medicina, Universidade de Brasília, Brasília 70910-900, DF, Brazil; 5 Instituto Nacional de Ciência e Tecnologia e Núcleo de Biologia Estrutural e Bioimagens—CENABIO, Rio de Janeiro 21941-902, RJ, Brazil

**Keywords:** cardanol derivatives, phospholipid analog, neglected disease, Chagas disease, new compounds

## Abstract

**Background/Objectives:** Chagas disease, caused by *Trypanosoma cruzi*, remains a major neglected tropical disease with limited therapeutic options restricted to benznidazole and nifurtimox, both associated with significant toxicity and reduced efficacy during chronic infection. Seeking novel, safe, and sustainable chemotherapeutic candidates, two new saturated cardanol-derived phospholipid analogs—LDT10 and LDT119—were rationally designed based on the molecular scaffold of miltefosine and biosourced from cashew nut shell liquid (CNSL). This study aimed to evaluate the pharmacokinetic properties of these compounds in silico and assess their antiparasitic activity, cytotoxicity, and morphological and ultrastructural effects on all developmental forms of *T. cruzi* in vitro. **Materials and Methods:** In silico ADMET predictions (SwissADME, pkCSM) were performed to determine bioavailability, pharmacokinetic behavior, CYP inhibition, mutagenicity, and hepatotoxicity. Antiproliferative activity was evaluated in epimastigotes, trypomastigotes, and intracellular amastigotes using dose–response assays and flow cytometry. Cytotoxicity was assessed in HEPG2 and HFF-1 cells using resazurin-based viability assays. Morphological and ultrastructural alterations were investigated through scanning (SEM) and transmission (TEM) electron microscopy. Reactive oxygen species (ROS) generation was quantified with H_2_DCFDA after 4 h and 24 h of exposure. **Results:** In silico analyses indicated favorable drug-like profiles, high intestinal absorption (>89%), absence of mutagenicity or hepatotoxicity, and non-penetration of the blood–brain barrier. LDT10 was not a P-gp substrate, and LDT119 acted as a P-gp inhibitor, suggesting reduced efflux and higher intracellular retention. Both compounds inhibited epimastigote proliferation with low IC_50_ values (LDT10: 0.81 µM; LDT119: 1.2 µM at 48 h) and reduced trypomastigote viability (LD_50_ LDT10: 2.1 ± 2 µM; LDT119: 1.8 ± 0.8 µM). Intracellular amastigotes were highly susceptible (IC_50_ LDT10: 0.48 µM; LDT119: 0.3 µM at 72 h), with >90% inhibition at higher concentrations. No cytotoxicity was observed in mammalian cells up to 20 µM. SEM revealed membrane wrinkling, pore-like depressions, rounded cell bodies, and multiple flagella, indicating cell division defects. TEM showed Golgi disorganization, autophagic vacuoles, mitochondrial vesiculation, and abnormal kinetoplast replication, while host cells remained structurally preserved. Both compounds induced significant ROS production in trypomastigotes after 24 h in a dose-dependent manner. **Conclusions:** LDT10 and LDT119 exhibited potent and selective in vitro activity against all developmental stages of *T. cruzi*, with low micromolar to submicromolar IC_50_/LD_50_ values, minimal mammalian cytotoxicity, and extensive morphological and ultrastructural damage consistent with disruption of phospholipid biosynthesis pathways. Combined with favorable in silico pharmacokinetic predictions, these CNSL-derived phospholipid analogs represent promising candidates for future Chagas disease chemotherapy and warrant further in vivo evaluation.

## 1. Introduction

Chagas’ disease is an infectious disease caused by the parasite *Trypanosoma cruzi*. This is considered a neglected tropical disease (NTD) mainly caused by insect-borne protozoan parasites transmitted through the bite of infected insects (Triatominae, popularly known as “kissing bug”). Over 100,000 people die each year from NTDs worldwide, most living on less than USD 2 per day [1]. There are only two drugs available for treating Chagas disease: benznidazole (BZN) and nifurtimox (NFX). Moreover, the safety profile of the currently available drugs is far from ideal, with frequent adverse events and high drug discontinuation rates [2,3]. Although these drugs are effective in the acute phase, they have limitations for chronic infections, and both drugs are restricted due to their toxic effects. For example, Nifurtimox is no longer used in many places since it causes neurological disorders or psychiatric episodes. Therefore, there is an urgent need to develop effective, safe, and affordable drugs [4]. Although the exact mechanism underlying benznidazole’s activity is unknown, it is activated by NADH-dependent trypanosomal reductases. It produces reductive metabolites that are believed to have several adverse consequences, including DNA damage and inhibition of protein synthesis [5]. Faced with this scenario, various strategies for new treatment are under investigation, including drug repurposing, combination therapies, and the development of new compounds [6,7]. Antifungal agents (posaconazole) failed to achieve parasitological cure in clinical trials [8,9]. Fexinidazole, an analog of BZN, has emerged as a potential alternative, though its clinical superiority remains unproven [10]. Concurrently, treatment optimization efforts aim to reduce adverse effects and improve adherence, with shorter regimens showing promise in minimizing toxicity without compromising efficacy [11]. However, key challenges persist, including the lack of reliable biomarkers for monitoring therapeutic response and limited funding for large-scale trials. Thus, expanding access to new therapeutic compounds is essential.

One of the strategies that has gained prominence in this decade is the development of ideal drugs, known as One Health Drugs, that simultaneously contribute to the health and sustainability of humans, animals, and the environment. In this sense, the use of phenolic lipids found in cashew nut shell liquid (CNSL) from *Anacardium occidentale*—natural or technical as a by-product of the food industry—is a strategic opportunity for the rational design of novel candidates for sustainable and low-cost therapeutic agents. Several studies on the biological [12,13,14] and chemical [15,16] profiles of the phenolic lipids of CNSL, in the form of their mixtures or isolated compounds or even as semi-synthetic derivatives, describe phenotypic and bioreceptor actions, including antioxidant [17,18] anti-inflammatory [19,20] antinociceptive [21], antimicrobial [22,23,24], larvicidal [25], and antiparasitic [26,27] activities. Based on these facts and considering that phospholipid analogs can inhibit the development of diseases caused by trypanosomatids such as *Leishmania amazonensis*, where phospholipid analogs, such as miltefosine, cause apoptosis, we aimed to assess the in vitro activity of some new CNSL against *T. cruzi*. Structurally, the phenolic lipid mixtures of CNSL, e.g., anacardic acids, cardanols, cardols, and 2-methylcardols, have at least a polar hydroxyl group and an apolar alkyl pentadecyl chain that can be saturated or unsaturated (monoene, diene, and triene) attached to the aromatic ring. These features constitute a natural biophoric system with electronic and lipophilic properties relevant to molecular recognition by different therapeutic targets [28]. The new compounds were designed based on the molecular scaffold of miltefosine, retaining the lipophilicity of the hexadecyl group identified in the pentadecyl chain of the saturated cardanol (LDT10). In turn, the phosphate group of the phosphocholine subunit was replaced by the aromatic ring and the choline ether by a protonable *N*-ethylpiperidine group linked directly to the oxygen of the phenol, yielding the new compound 1-(2-(3-pentadecylphenoxy)ethyl)piperidine (LDT119). Here, we observed that both new compounds were able to act against all three forms of *T. cruzi* more effectively than the compound of choice, benznidazole. LTD10 and LDT119 inhibited the growth of intracellular amastigotes with an IC50 of 0.48 and 0.3 μM, respectively. SEM observations revealed the presence of protrusions on the surface after treatment. TEM analyses revealed vacuoles containing myelin figures and cytoplasmic vacuolation after incubation with the compounds. Furthermore, after treatments with the compounds LDT10 and LDT119 the parasites showed alterations in mitochondrial activity and ROS production. Our observations indicate that the compounds tested showed promise against *T. cruzi* in vitro, suggesting their potential usefulness as an alternative chemotherapy for Chagas disease.

## 2. Results

### 2.1. In Silico Analysis

**Figure 1:**
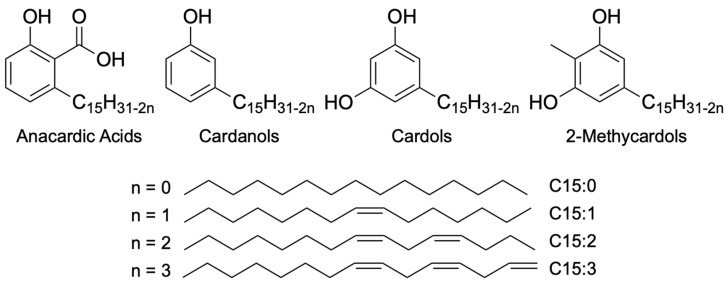
Structures of CNSL constituents.

**Figure 2:**
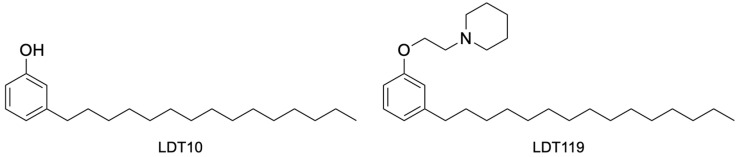
LDT10 and LDT119 structure.

The two new compounds produced from CNSL (Figure 1 and Figure 2) were analyzed for their pharmacological properties using the SwissADME and pkCSM programs, the results were compiled and presented in Table 1, organized by evaluated properties, for better understanding and comparison. The compound LDT10 is not a substrate of gp-P and, consequently, is not its inhibitor. In contrast, LDT119 exhibits P-gp inhibitory activity. Therefore, it can be inferred that the LDT compounds of this study, due to their pharmacokinetic characteristics, will not be easily expelled by the parasite or host cells of intracellular amastigotes. In addition, these characteristics also predict a more efficient therapeutic effect, which, according to the studies cited above, will not lead to the protozoan developing resistance to treatment, thereby being beneficial for both the acute and chronic phases of the disease.

The blood–brain barrier (BBB) is a crucial component of Central Nervous System (CNS) homeostasis, serving as a protective mechanism for drug entry into the CNS. In this context, LDT10 and LDT119, as indicated by the results obtained in this assay, are unable to cross the natural protections in the CNS.

**Figure 3:**
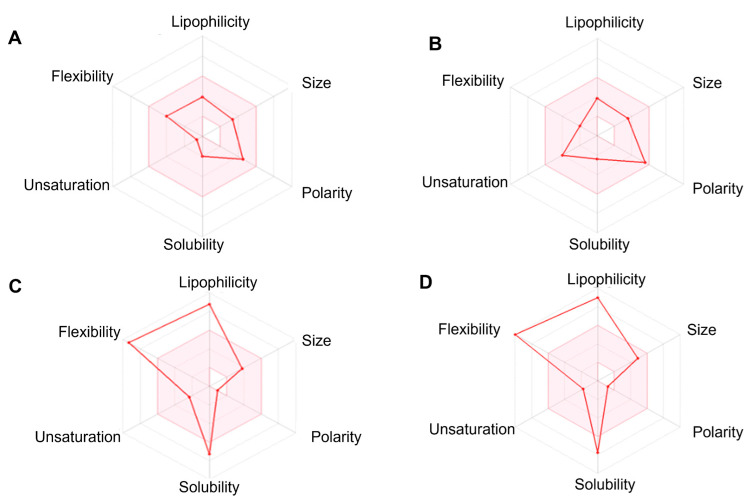
The bioavailability radar of compounds is acquired from the evaluation of molecular structures in SwissADME. (**A**) Benznidazole; (**B**) Nifurtimox; (**C**) LDT10; (**D**) LDT119. Note that only commercial drugs showed optimal bioavailability, with all points on the graph within the pink area.

When submitted to SwissADME’s bioavailability analyses, the compounds generally presented size, unsaturation, and polarity within the ideal range, and the liposolubility, flexibility, and solubility in water were slightly distant from what the program defined as ideal under the assumed parameters. The commercial drugs BNZ and NTX, when calculated using the same parameters and plotted on the bioavailability radar, presented acceptable standards within the defined ideal range (Figure 3A and Figure 3B, respectively).

Given a generic overview of the six physicochemical properties plotted in the SwissADME Bioavailability Radar, three are outside the desired range (liposolubility, flexibility, and solubility in water), and three of them favor the therapeutic potential of the tested compounds (size, unsaturation, and polarity). Moreover, according to in silico predictions, they also lack properties that would allow them to permeate the BHE, exhibit high intestinal absorption, or demonstrate mutagenic or hepatotoxic potential. The LDT10 compound does not violate any of the criteria of Lipinski’s rule of five. As for the pharmacokinetic properties, the compounds, in general, present a homogeneous pattern; that is, few have very distinct properties of inhibition of the gp-P or even CYP isoenzymes. The LDT10 compound is not a substrate of gp-P, while LDT119 is an inhibitor of the enzyme gp-P. These two conditions (neither being a substrate nor inhibiting P-gp) would allow for higher intracellular concentrations of the two compounds, thereby targeting the trypomastigote and intracellular amastigote forms.

### 2.2. Effects of LDT Compounds on T. cruzi

**Figure 4:**
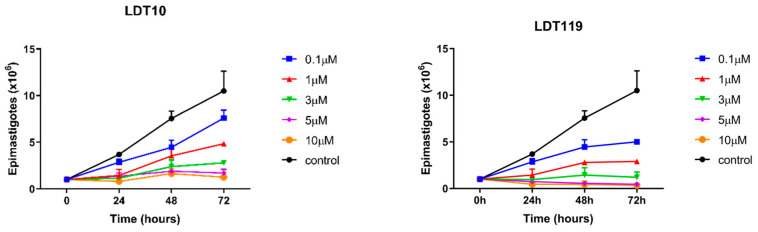
Dose–response curve of phospholipid analog compounds on the proliferation of *T. cruzi* epimastigotes. LDT10 curve: IC50 = 0.48 µM; LDT119 curve: IC50 = 0.30 µM. The values represent the means ± standard deviations (bars) of experiments performed in triplicate. Note that the compounds were effective against the proliferation of *T. cruzi* epimastigotes.

To observe the effect of LDT compounds on the proliferation of *T. cruzi* epimastigotes cultured in LIT (“liver infusion tryptose”) medium supplemented with 10% inactivated fetal bovine serum (FBS—Thermo LifeScience, Waltham, MA, USA) at a concentration of 2 × 10^6^ cells/mL. No antibiotics or antifungals were added. After 24 h, they were treated with 0.1 µM to 10 µM of LDT10 or LDT119 for 24, 48, and 72 h. Every 24 h, an aliquot of each condition was removed and counted using a hemocytometer and optical microscope (Figure 4).

These results demonstrate that the compounds were effective in inhibiting the proliferation of *T. cruzi* epimastigotes. From the curves obtained, the IC50 values for the proliferation of epimastigotes were calculated. Both treatments showed low IC50 values: 0.81 µM for the LDT10 treatment (48 h) and 1.2 µM for the LDT119 treatment (48 h). The treatment with LDT119 reduced the number of epimastigotes by more than 90% within the first 24 h after treatment, at concentrations above 5 μM. After 72 h of treatment, a decrease of more than 95% was observed at the same concentrations compared to the control. However, at no concentration was it possible to observe parasitological cure, where there are no more proliferative cells. When we observe the potential to reduce proliferation caused by treatment with LDT10, we note that the maximum reduction in epimastigote proliferation was 85% after 72 h of treatment compared to the control.

**Figure 5:**
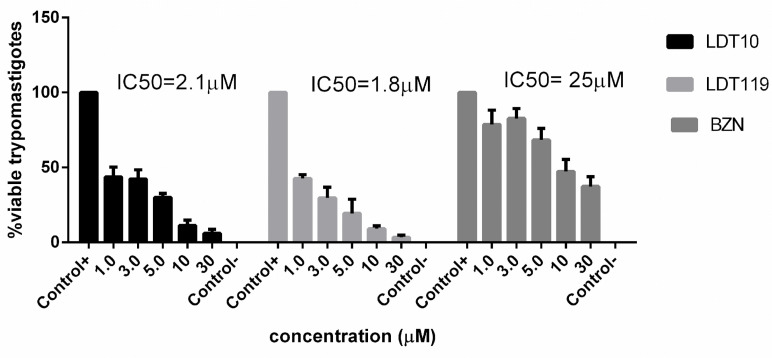
Flow cytometry analysis of viability in *T. cruzi* trypomastigotes by labeling with propidium iodide (PI). Treatment with LDTs induced permeabilization of the parasite’s plasma membrane and consequent loss of viability. As a positive control, the trypomastigotes were incubated with 0.1% saponin. The graphs show the average of four independent experiments, and the LD50 was determined by the percentage of viability in each concentration compared to the untreated control.

To analyze the effect of LDTs on the viability of trypomastigotes, they were treated with the compounds LDT10 and LDT119 for 2 h at concentrations of 1 μM, 3 μM, 5 μM, 10 μM, and 30 μM. The cells were incubated with propidium iodide (PI) for further analysis in flow cytometry. In all treatments, a loss of viability has already occurred at the lowest concentration, and the LD50 values are indicated in the graph (Figure 5). The LD50 values obtained were 2.1 ± 2 μM and 1.8 ± 0.8 μM for the compounds LDT10 and LDT119, respectively.

**Figure 6:**
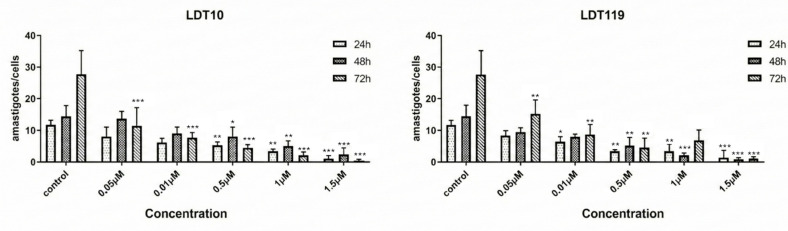
Proliferation of intracellular amastigotes in HFF-1 treated with LDT10 and LDT119. Both compounds reduced the number of intracellular amastigotes after 24 h of treatment and decreased the amastigote population by at least 50% at a concentration of 0.5 µM. The data represent the average of three independent experiments, each performed in triplicate. Values represent mean ± SD of 3 duplicate experiments. IC50 calculated for 72 h (LDT10—0.48 µM; LDT119—0.3 µM). * *p* < 0.05; ** *p* < 0.01; *** *p* < 0.005.

To observe the effect of LDT10 and LDT119 on amastigote proliferation, HFF-1 cells containing intracellular amastigotes were treated for 24, 48, and 72 h with concentrations of 0.05 μM, 0.01 μM, 0.5 μM, 1.0 μM, and 1.5 μM of each of the analogs. The number of amastigotes per host cell is shown in Figure 6. Treatment of intracellular amastigotes with LDT resulted in reduced proliferation, with a concentration-dependent effect. After 48 h of treatment, an IC50 of 0.48 μM can be obtained for the treatment of amastigotes with LDT10 and 0.3 μM for the treatment of amastigotes with LDT119. Both treatments promoted inhibition of amastigote proliferation at levels above 90% at the highest concentrations tested.

### 2.3. Evaluation of the Cytotoxicity of the Compounds in Cells of the HEPG2 and HFF-1 Strains

**Figure 7:**
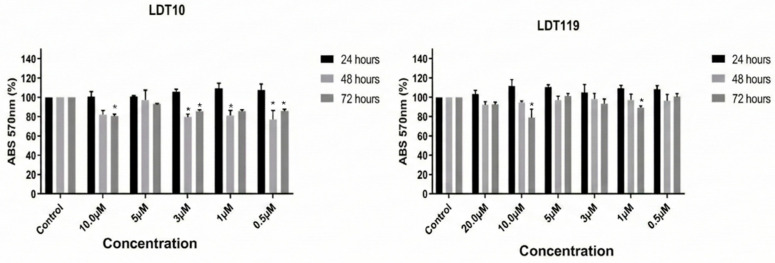
Hepatotoxicity of LDT compounds evaluated in cells of the HEPG_2_ lineage in 24, 48, and 72 h. Neither compound was able to cause the death of more than 25% liver cells, regardless of the concentration used. The data represent the average of three independent experiments, each performed in triplicate. Values represent mean ± SD of 3 duplicate experiments. * *p* < 0.05.

**Figure 8:**
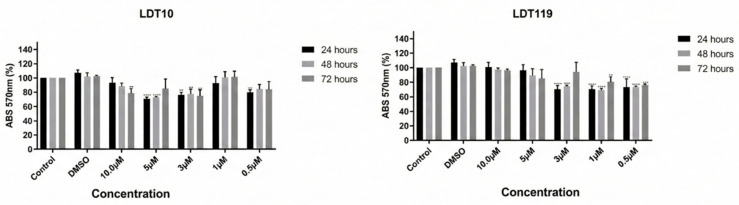
Cytotoxicity of LDT compounds evaluated in HFF-1 cells in 24, 48, and 72 h. Neither compound was able to cause the death of liver cells, regardless of the concentration used. The data represent the average of three independent experiments, each performed in triplicate. Values represent mean ± SD of 3 duplicate experiments.** *p* < 0.01; *** *p* < 0.005, **** *p* < 0.001.

The LDT’s cytotoxic effects in vitro were evaluated in mammalian cell lines, including HEPG2 to assess the risk of hepatotoxicity and HFF-1, a host cell for intracellular amastigotes. For this, both cell types were treated with increasing concentrations (1 μM to 20 μM) for up to 72 h. The results are presented in Figure 7 and Figure 8.

### 2.4. Effects of Phospholipid Analogs on the Morphology of Epimastigotes by Scanning Electron Microscopy (SEM)

**Figure 9:**
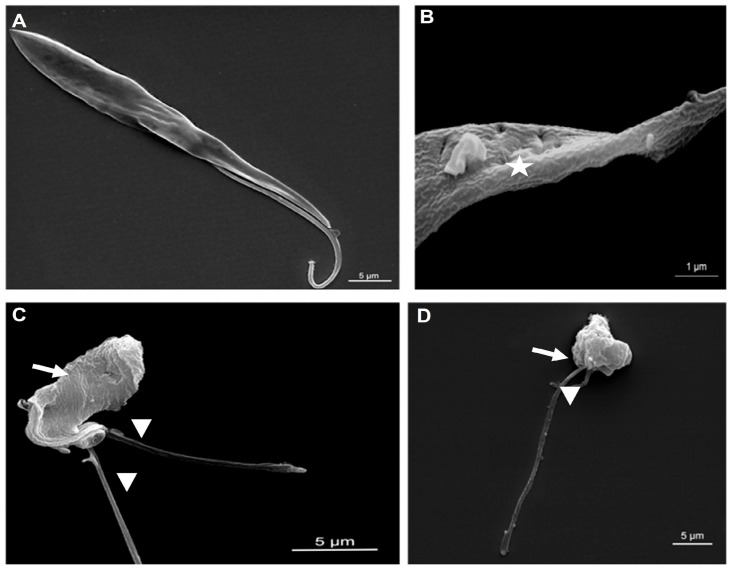
SEM of epimastigotes treated with LDTs for 48 h of treatment. (**A**) Untreated cells: elongated cell body and a single tapered flagellum. (**B**,**C**) Epimastigotes treated with LDT10 demonstrate changes in cell body morphology (arrow), multiple flagella (arrowhead), and pore-like depressions in the membrane (star). (**D**) Epimastigotes treated with LDT119, a rounded cell body (arrow) and multiple flagella (arrowhead).

To verify the effects of phospholipid analog compounds on the morphology and structure of epimastigotes of *T. cruzi*, the compounds LDT10 and LDT119 were used for the analysis of cell morphology by scanning electron microscopy. The treatment concentrations used were 1 μM for the compound LDT119 and 1.5 μM for the compound LDT10 when epimastigotes were treated for 48 h (Figure 9). Wrinkling of the plasma membrane of the epimastigotes can also be observed (Figure 9B–D), with the presence of pore-like depressions in the membrane (Figure 9B) and more than one flagellum without division of the cell body (Figure 9C,D), indicating cell-cycle problems. Note also the rounded cell body (Figure 9C,D) when compared with the control (Figure 9A). It was observed that approximately 65% of the analyzed cells exhibited problems related to cell division, with cells formed by several flagella.

### 2.5. Analysis of the Effect on Ultrastructure of Epimastigotes and Amastigotes of T. cruzi Treated with LDT Compounds

**Figure 10:**
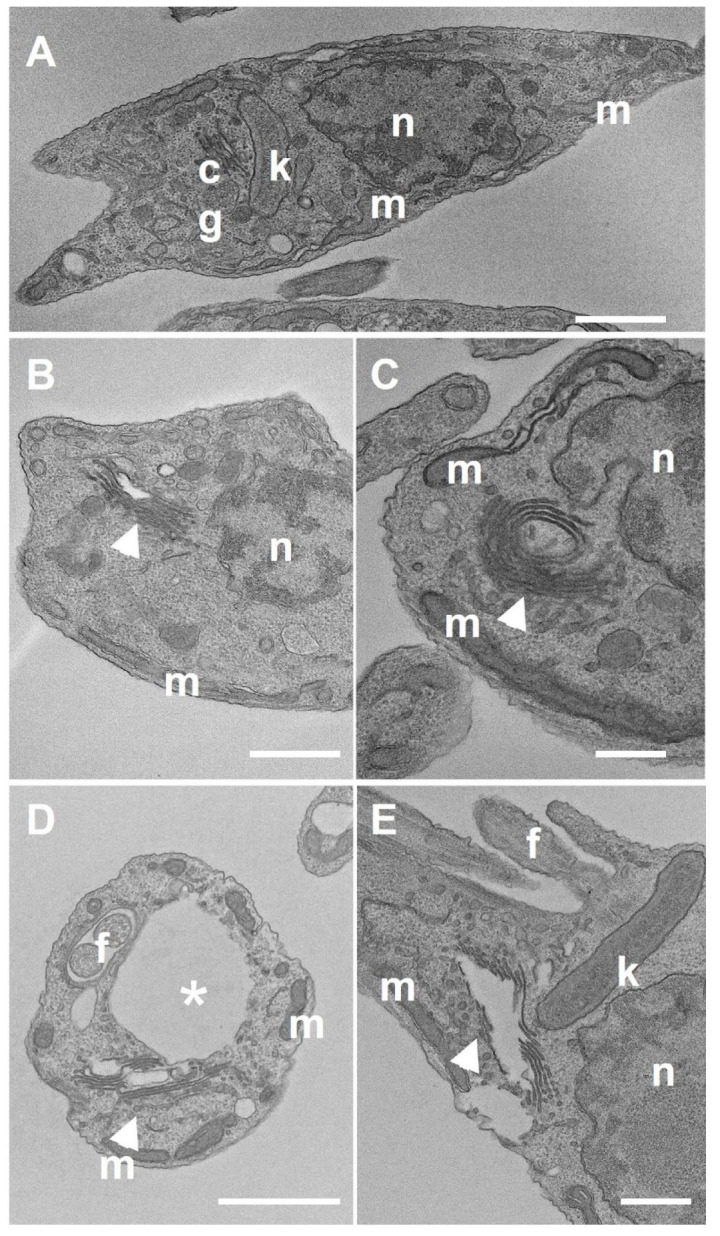
Transmission electron microscopy of epimastigotes treated with LDTs for 48 h. (**A**) Control without treatment shows all structures preserved. (**B**,**C**) Epimastigotes treated with LDT10 display a misorganization of the Golgi complex (arrowhead). (**D**,**E**) Epimastigotes treated with LDT119 also demonstrate a poor organization of the Golgi complex (arrowhead) and the presence of vacuoles (asterisk). n = nucleus; k = kinetoplast; cg = Golgi complex; f = flagellum; m = mitochondria. Bars = 0.5 μm.

Transmission electron microscopy was used to investigate the effects of LDT compounds on the ultrastructure of *T. cruzi*. For epimastigotes, the same concentrations and times used for scanning electron microscopy were used (Figure 10). Without any treatment, epimastigote forms present a single nucleus, containing the condensed heterochromatin near the nuclear envelope and around the nucleolus, the bar-shaped kinetoplast near the flagellum, and a single branched mitochondrion. They are characterized by the elongated shape of the cell body (Figure 10). All treatments with the compounds were able to induce similar ultrastructural changes. After treatment, changes in the Golgi complex (Figure 10B,C,E—arrow) and formation of vacuoles similar to autophagic vacuoles (Figure 10D—asterisk).

**Figure 11:**
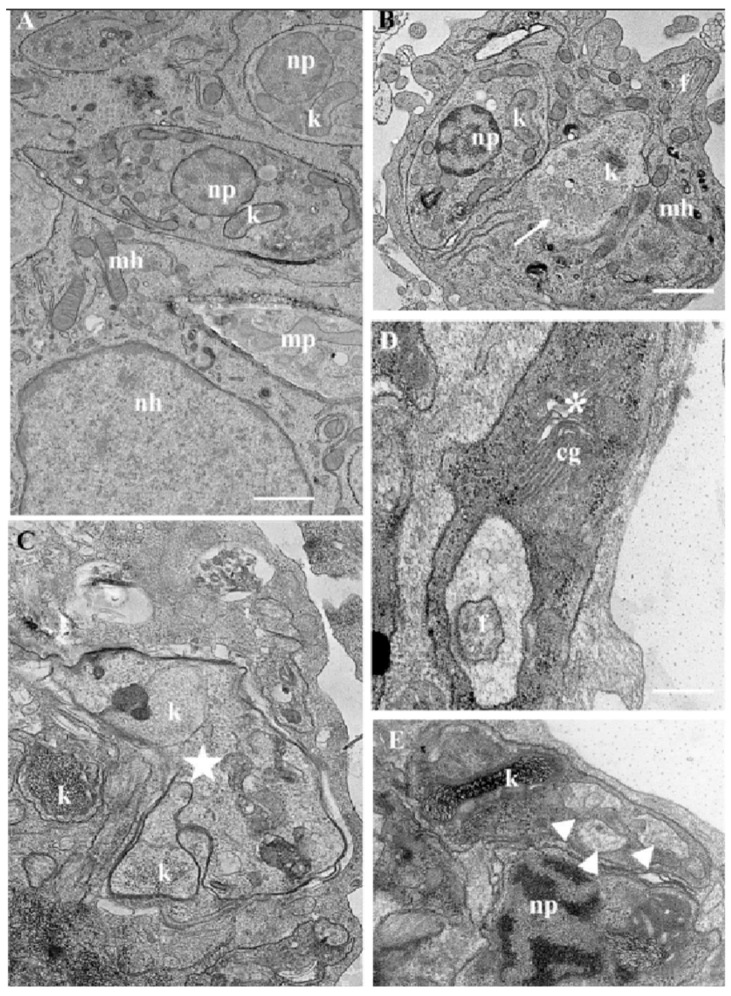
Transmission electron microscopy of intracellular amastigotes treated with LDTs for 48 h. (**A**) Control without treatment preserves all structures in both the host cell and the parasites. (**B**,**C**) Intracellular amastigotes treated with LDT10, the presence of intact *T. cruzi* and partially degraded parasites (indicated by the white arrow), as well as multiple kinetoplasts within the same cell body (k—white star), indicating problems with cell division, is observed. (**D**,**E**) Intracellular amastigotes treated with LDT119 demonstrate poor organization of the Golgi complex (cg—asterisk) and formation of mitochondrial vesicles (arrowhead). Note: np = parasite nucleus; nh = host cell nucleus; k = kinetoplast; cg = Golgi complex; mp = mitochondria; f = flagellum; mh = mitochondria of the host cell. Bars = 0.5 μm.

The ultrastructural modifications of intracellular amastigotes after 48 h of treatment with LDT10 and LDT119 (IC50) demonstrated the presence of vacuoles similar to autophagic vacuoles inside the parasites (Figure 11B—arrowhead). Another alteration in the ultrastructure commonly observed was the presence of parasites with multiple kinetoplasts and poorly compacted kDNA, a characteristic of the kinetoplast of trypomastigotes (Figure 11C,D). This is a clear indication of cell cycle problems. The host cells did not exhibit any changes in their ultrastructure.

### 2.6. ROS Production

**Figure 12:**
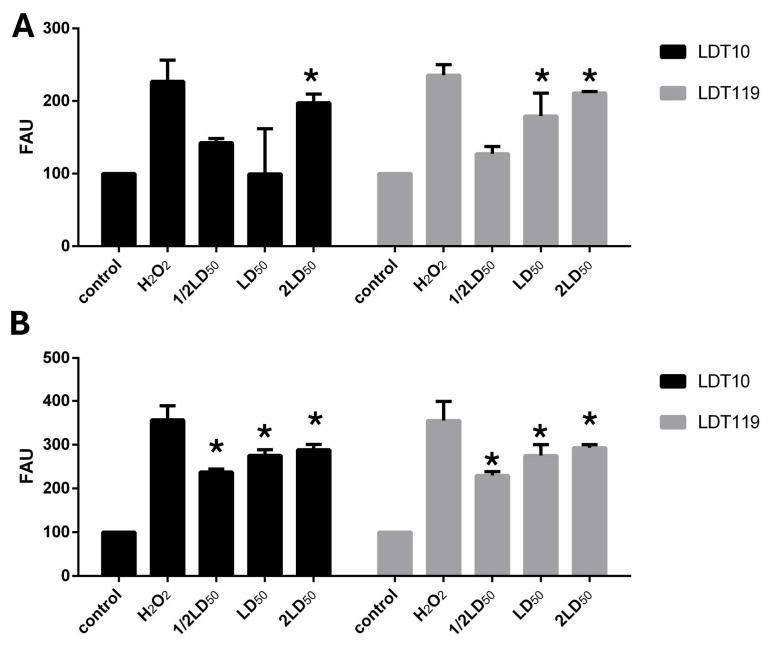
The production of ROS was measured fluorometrically in control cells and those treated with ½ × LD50 (1 µM), LD_50_ (2 µM), and 2 × LD50 (4 µM) for 4 h (**A**) and 24 h (**B**), using the green fluorescent probe H_2_DCFDA. Cells treated with 1 mM H_2_O_2_ were used as a positive control for the intracellular generation of ROS. The results are expressed as fluorescence arbitrary units (FAU). Values represent mean ± standard deviation of three independent experiments. The asterisks represent significant statistical differences compared to the respective controls (*p* < 0.01).

The ability of LDT10 and LDT119 to induce oxidative stress was also investigated using the probe H_2_DCFDA. Trypomastigotes exposed to both compounds for only 4 h showed a slight but significant production of ROS, only at their approximately 2 × LD50 value (4 µM), the highest concentration used (Figure 12A). However, after 24 h of drug contact, the oxidative stress generated rose significantly in a dose-dependent manner (Figure 12B). At the LD50 doses (2 µM), both compounds increased ROS production by 47% and 56%, respectively. Cells treated with H_2_O_2_ served as positive controls and exhibited high fluorescence levels (Figure 12).

## 3. Discussion

The biosynthesis of phospholipids is already a recognized target for drug development, given the crucial role these molecules play in cell structure and function. Phospholipid analogs are a new class of pharmaceutical products that demonstrate excellent biocompatibility and unique amphiphilicity, making them suitable for use as drugs with broad-spectrum biological activity. Over the past few decades, the overall demand for synthetic phospholipids, primarily for use in drug development processes, has increased [29]. PAs can be subdivided into two classes—alkylglycerophosphocholines, derived from edelfosine (ET-18-OCH_3_), and alkylphosphocholines, derived from miltefosine (MLT) (hexadecylphosphocholine). Both classes act to inhibit the synthesis of phosphatidylcholine [30]. APLs can be natural and synthetic [31]. In recent years, the demand for synthetic phospholipids has increased, prompting the development and testing of new synthetic routes for their production over several years. In the late 60 s, the first phospholipid derivative was synthesized, known as edelfosine (1-octadecyl-2-O-methyl-rac-glycero-3-phosphocholine). Edelfosine was initially evaluated as an antitumor compound for its immunomodulatory effect and its ability to inhibit tumor cell proliferation. In addition to its antitumor activity, edelfosine has demonstrated efficacy against Leishmania spp., both in vitro and in vivo in infected mouse models [29].

The effectiveness of edelfosine against Leishmania is related to the fact that the growth and proliferation of parasites depend on their ability to recruit molecules essential for membrane assembly. It is known that parasites of the order Kinetoplastida capture lipids from their hosts for the formation of membranes. However, genetic, biochemical, and pharmacological data have made it clear that de novo lipid synthesis plays a crucial role in the viability of the parasite at different stages of its life cycle [32].

In this study, in silico evaluations were initially performed. Such preliminary assays involved analyses of the compounds’ pharmacokinetic properties, bioavailability, mutagenic and hepatotoxic potential, and the permeability capacity of the blood–brain barrier. In this context, the analysis in silico of pharmacokinetic properties carried out in this research allowed us to identify compounds with an inhibitory capacity of the enzymes of the cytochrome P450 enzyme complex, knowing that the enzymes CYP1A2, CYP2C9, CYP2C19, CYP2D6, and CYP3A4 metabolize 90% of the drugs, the last two with greater prominence. These enzymes are predominantly expressed in the liver but also occur in the small intestine (reducing the bioavailability of drugs), lungs, placenta, and kidneys [33].

Therefore, the compounds evaluated by in silico analysis that demonstrate the inhibitory potential of a greater number of isoforms of CYP enzymes will provide the patient with a higher concentration of this compound in their cells, the opposite being also true, that is, the lower the number of CYP the compound inhibits, the higher the concentration administered for there to be an expected effect. However, it is worth mentioning that different concentrations of a compound can cause unexpected effects on the patient, ranging from the absence of therapeutic responses to toxicity and, according to Katsuno and colleagues [34], the ideal is that new compounds do not present characteristics of inhibition of CYP, otherwise, there may be toxic adverse effects or other undesirable adverse effects, due to the lower clearance and accumulation of the drug or its metabolites in the host’s body [35].

Then, the administered concentration and the plasma concentration (absorbed in the mesentery) should be studied. Based on this information, the maximum dose suggested for the compounds can be used to start in vivo assays, for example [36]. In silico analyses showed that the LDT compounds tested are not able to inhibit all isoenzymes of the CYP450 family, and this is a positive characteristic, in favor of the potential use of these compounds as future drugs, since, according to Tornio and Backman [37], even if some enzymes are inhibited, others can be very effective in metabolizing exogenous com-pounds, thus avoiding significant adverse effects. Nevertheless, in vitro evaluations of 15 different isoenzymes of the CYP450 family were tested for miltefosine (marketed for the treatment of leishmaniasis in India), and none of them showed oxidative metabolism for this molecule. Some studies have shown that, most likely, miltefosine is metabolized intracellularly by phospholipase D, rather than by CYP [38,39]. It should also be considered that the degradation products of miltefosine are abundant endogenous compounds and are, therefore, difficult to quantify. For example, choline is involved in the biosynthesis of cell membranes. There is little excretion of unchanged miltefosine. Some studies indicate that the excretion of miltefosine in urine accounts for only ≤ 0.2% of the administered dose on day 23 of treatment [40,41].

Another site considered essential for the biotransformation of drugs is the intestinal mucosa, where a significant amount of P-gp is present [41], responsible for the efflux of xenobiotics. The role played by membrane transporters in the absorption, disposition, and excretion of compounds has been increasingly recognized. In particular, P-glycoprotein (P-gp; encoded by the MDR1 or ABCB1 gene in humans) has demonstrated a significant impact on pharmacokinetics of compounds, limiting oral absorption, restricting CNS penetration, and promoting excretion. Thus, as stated earlier, a compound that is the substrate of this glycoprotein will be carried and excreted by the body more quickly, unless it exerts an inhibitory function on the same. The latter will tend to increase its intracellular concentration, resulting in greater therapeutic responses and, possibly, more severe adverse or cytotoxic effects. However, studies have shown that drug resistance in protozoan parasites has been associated with P-gp [42]. Previous studies, such as those by Murta [43], had already reported resistance of *T. cruzi* to treatment with BNZ and NTX.

Thus, it is understood that P-gp inhibitors improve the absorption of compounds, while P-gp substrates reduce their absorption [40]. P-gp substrates can be easily pumped out of cells, reducing their absorption; conversely, P-gp inhibitors may inhibit this pumping activity. Therefore, determining whether a compound is a P-gp substrate or an inhibitor becomes critical in the early stages of developing new chemotherapy compounds [44].

Among the LDT compounds that underwent in silico analysis in SwissADME for this parameter, we have LDT10, which is not a substrate of gp-P and, consequently, is not an inhibitor of it. In contrast, the compound LDT119 exhibits P-gp inhibitory activity. Therefore, it can be inferred that the LDT compounds of this study, due to their pharmacokinetic characteristics, will not be easily expelled by the parasite or by the host cells of intracellular amastigotes. In addition, these characteristics also predict a more efficient therapeutic effect, which, according to the studies cited above, will prevent the protozoan from developing resistance to treatment, thereby benefiting both the acute and chronic phases of the disease.

BHE expresses influx and efflux transporters that precisely control the permeation of circulating solutes, including drugs. For some compounds, depending on their purpose of action, it is crucial that they can permeate the BHE and act on CNS cells, as in the therapy of Alzheimer’s, Parkinson’s, and even African Trypanosomiasis, for example [45]. In other cases, when it is not intended to reach the CNS, drug candidates shouldn’t be able to permeate the BHE, thus reducing the amount of possible adverse effects. Geldenhuys et al. [45] discussed the importance of this prediction, highlighting that many research projects involving compounds with therapeutic potential can be interrupted during clinical trials simply because they do not perform as well or better than existing compounds on the market. After all this information regarding the permeability of BHE was deprecated from the beginning of the experiments. Few BHE permeability articles from companies address this issue, which should be encouraged as a matter of primary interest. Therefore, in this research, the compounds were subjected to such analyses. As a result, not even the drugs of choice (BNZ and NTX) can cross this natural CNS protection. Therefore, it can be inferred from these data that LDT compounds have no effects in the CNS, reducing the possibility of causing adverse effects in future patients.

In the in vitro assays, epimastigote forms of *T. cruzi* were used to screen the compounds, determine the IC50, and consequently identify the most effective compound in killing the parasites. Then, in vitro tests were performed using trypomastigote forms (non-reproductive, infective form) and intracellular amastigote to verify the compounds’ effect and their ability to reduce the viability and proliferation of developmental forms in the host organism. In the assays performed in trypomastigotes, loss of viability was observed from the lowest concentration, and the LD50 values were close to those obtained in epimastigotes in 24 h. The LD50 values obtained were 2.1 ± 2 μM and 1.8 ± 0.8 μM for compounds LDT10 and LDT119, respectively. On the other hand, treatment of intracellular amastigotes with LDT showed reduced proliferation and a dose-dependent effect. The IC50 was calculated from the data obtained in 72 h. Comparing the results with those obtained on trypomastigotes, the IC50 values (24 h) were much lower than the LD50 values obtained in the same treatment time: 0.63 ± 0.45 μM and 0.62 ± 0.13 μM for the compounds LDT10 and LDT119, respectively. This suggests that the compounds are more effective against intracellular amastigotes than against other stages of *T. cruzi* development. These results corroborate the promising potential of the selected phospholipid analog compounds, consolidating what had already been predicted in the in silico analyses.

The in vitro cytotoxic effects of LDT were evaluated in mammalian cells, specifically HEPG_2_ (to assess the risk of hepatotoxicity) and HFF-1 (a host cell for intracellular amastigotes). In the in silico assays, the compounds did not show hepatotoxic potential, and when the in vitro tests are compared, the LD50 and IC50 concentrations obtained trypomastigotes and amastigotes, respectively. The concentrations used in the assay with the HEPG_2_ strain reveal a discrepancy, leading to the conclusion that the concentrations used to reduce the viability of trypomastigotes and inhibit the proliferation of intracellular amastigotes of *T. cruzi* will not significantly affect liver cells. The same applies to the HFF-1 strain; that is, the concentrations of LD50 and IC50 for trypomastigotes and intracellular amastigotes of *T. cruzi*, respectively, will not significantly reduce the viability/proliferation of host cells. However, more advanced tests, such as in vivo tests, can be performed to confirm this hypothesis and evaluate possible adverse effects.

Many different phospholipid analogs have already been tested on trypanosomatids. One of the first compounds developed and evaluated after edelfosine was ilmofosine. The in vitro activity of ilmofosine against *L. donovani* amastigotes with IC50 = 3.7 μM and against antimony-resistant *L. infantum* amastigotes with IC50 = 3.5 μM [46]. In addition, ilmofosine was effective against amastigotes of *T. cruzi* (strain Y) in murine macrophages with an IC50 < 2.0 μΜ. Additional studies against intracellular epimastigotes and amastigotes in the heart muscle or Vero cells have demonstrated the high efficacy of ilmofosine. However, the activity against *T. b. brucei* and *T. b. rhodesiense* was significantly lower [46]; however, miltefosine, despite serious side effects, including hemolysis and cumulative gastrointestinal toxicity [47]. In general, the IC_50_ for perifosine treatment in promastigotes of different Leishmania species ranges from 2 μM to 15 μM [48]. In intracellular amastigotes, the IC50 after treatment with this compound is less than 5 μM [49].

Regarding *T. cruzi*, our group demonstrated that ether-phospholipid derivatives were significantly effective against *T. cruzi* in the nanomolar range when used against epi-mastigotes, trypomastigotes, or intracellular amastigotes [50,51]. It should be noted that, in vitro, all analogs tested on *T. cruzi* are more effective than the compounds of choice. In addition, when compared with the compounds already described in the literature, the compounds tested in the present study (LDT10 and LDT119) are about twice as efficient as the other phospholipid analogs.

Regarding the morphological changes observed by scanning electron microscopy (SEM), all are consistent with the descriptions of the possible effects of alkyl phospholipid-type compounds, which can reduce the biosynthesis of phosphatidylcholine, interfering with the standard formation of the plasma membrane. This process can interfere with the “lipid rafts”, responsible for anti-apoptotic signals, thus inducing stress in the transcription of pro-apoptotic factors; cause cell lysis by detergent/surfactant action, or even accumulate in the plasma membrane and interfere with signal transduction and phospholipid biosynthesis [52].

The changes in cellular ultrastructures observed by TEM in assays with LDT10 and LDT119 ratify what is described in the literature about the possible effects of phospholipid-analogous compounds. These compounds can induce the formation of autophagic vacuoles and autolysosomes, which trigger autophagy processes and increase the formation of autophagic vesicles that do not complete the fusion process with the lysosome. They also reduce the production of phosphatidic acid and diacylglycerol, regulators of specific functions of the Golgi Complex and secondary messengers in the chain of enzymes involved in cell proliferation. Specifically, in trypanosomatids, treatments with compounds similar to phospholipids, as reported in the literature, lead to modifications in the plasma membrane of the parasite body, changes in flagellar structure, alterations in mitochondria and the Golgi complex, and the presence of autophagic and lipid vacuoles [50,51,53]. The ultrastructural analysis also suggested that the specific mechanism of action of the phospholipid analogs (PAs) might be due to interference in two distinct phospholipid biosynthesis pathways used in the different cell types. Although it is not certain which pathway is affected, the observed effects suggests inhibition in Phosphatidylcholine biosynthesis (PAs like miltefosine and edelfosine, which the new compounds were designed based on, are known to act by inhibiting the synthesis of phosphatidylcholine) and inhibition of a pathway involving the production of phosphatidic acid and diacylglycerol (these molecules are regulators of specific functions of the Golgi Complex and secondary messengers in the chain of enzymes involved in cell proliferation) since the compounds affected the Golgi complex of the parasites, but not that of the host cells, suggesting interference in this pathway.

A general analysis of the results obtained in this research reveals that LDT compounds demonstrate significant potential, as evidenced by in vitro trials and in silico studies. This potential is also observed for the compounds evaluated at all stages, including the coupling simulation.

## 4. Materials and Methods

### 4.1. Synthesis of the LDT Compounds

1. 3-Pentadecylphenol (LDT10)

LDT10 (saturated cardanol) was obtained according to the protocol described by Sahin et al. [54]. The synthesis of saturated cardanol (LDT10), a phenolic lipid derivative, was achieved starting from a mixture of natural or technical grade cashew nut shell liquid (CNSL) constituents, specifically the cardanol mixture. The catalytic hydrogenation of the unsaturated chains present in the cardanol mixture resulted in the saturated cardanol.

2. (1-(2-(3-pentadecylphenoxy)ethyl)piperidine) (LDT119)

LDT119 was obtained in two steps from the compound LDT10 through the bromo intermediate LDT117 as described below:

3. 1-(2-bromoethoxy)-3-pentadecylbenzene derivative (LDT117)

To a 125 mL flask, 2.00 g of LDT10 (18) (6.57 mmol), a 10% sodium hydroxide solution (5.0 mL), six drops of Aliquat, and tetrahydrofuran (THF) (3.0 mL) were added. The mixture was subjected to magnetic stirring for 25 min to facilitate the formation of the phenolate ion, followed by the addition of 4.5 mL of 1,2-dibromoethane (52.54 mmol). The reaction mixture was refluxed at 70 °C, under magnetic stirring, for approximately 20 h. After this period, the solution was cooled in a water/ice bath and acidified with 50% hydrochloric acid until it reached a pH of 1.0. Then, the mixture was extracted with chloroform (three times, 20.0 mL), and the combined organic fractions were washed with a saline solution (20.0 mL) and dried over sodium sulfate. The solvent was evaporated under reduced pressure, and the residue was purified on a silica gel column using a dichloromethane-hexane mixture (1:1), which provided the LDT117 derivative as a white solid. Yield 90% (1.20 g, 2.92 mmol). m.p. 34.0–36.0 °C. 1H NMR (300 MHz, CDCl3): δ 0.91 (t, J = 6.0 Hz, 3H, 15′); 1.28 (sl, 24H, 3′–14′); 1.60 (t, J = 6.8 Hz, 2H, 2′); 2.60 (t, J = 7.6 Hz, 2H, 1′); 3.65 (t, J = 6.0 Hz, 2H, b); 4.30 (t, J = 6.0 Hz, 2H, a); 6.73–6.77 (m, 2H, 2 e 4); 6.83 (d, J = 7.5 Hz, 1H, 6); 7.20 (t, J = 7.6 Hz, 1H, 5). 13C NMR (75 MHz, CDCl3): δ 14.3 (CH3, 15′); 22.9 (CH2, 14′); 29.4–29.8 (CH2, 3′–12′); 29.9 (CH2, b); 31.6 (CH2, 2′); 32.2 (CH2, 13′); 36.2 (CH2, 1′); 68.0 (CH2, a); 111.9 (CH, 6); 115.3 (CH, 2); 121.9 (CH, 4); 129.5 (CH, 5); 145.1 (C, 3); 158.3 (C, 1) [54].

4. 1-(2-(3-pentadecylphenoxy)ethyl)piperidine (LDT119)

To a reactor tube (Ace pressure tube) were added 0.35 g of LDT117 (0.85 mmol), piperidine (2.04 mmol), triethylamine (2.04 mmol), and acetonitrile (0.3 mL). The reaction system was subjected to microwave radiation in a domestic oven for 5 min (5 × 1′) at 50% power [54]. The mixture was transferred to a silica gel chromatographic column and eluted with a chloroform-ethanol mixture, with the ethanol gradient increasing according to the polarity of each product, yielding derivative LDT134 as a yellow oil. Yield 85% (0.72 g, 1.7 mmol). 1H NMR (500 MHz, CDCl3): δ 0.89 (t, J = 6.8 Hz, 3H, 15′); 1.26–1.31 (m, 24H, 3′–14′); 1.46 (sl, 2H, e); 1.62–1,65 (m, 6H, 2′ e d); 2.56–2.59 (m, 6H, 1′ e c); 2.80 (t, J = 6.0 Hz, 2H, b); 4.12 (t, J = 6.1 Hz, 2H, a); 6.73 (d, J = 8.3 Hz, 1H, 2); 6.75 (s, 1H, 4); 6.77 (d, J = 7.6 Hz, 1H, 6); 7.18 (t, J = 7.8 Hz, 1H, 5). 13C NMR (75 MHz, CDCl3): δ 14.2 (CH3, 15′); 22.7 (CH2, 14′); 24.2 (CH2, e); 25.9 (CH2, d); 29.4–29.6 (CH2, 3′–12′); 31.4 (CH2, 2′); 32.0 (CH2, 13′); 36.1 (CH2, 1′); 55.1 (CH2, c); 58.0 (CH2, b); 65.7 (CH2, a); 111.5 (CH, 6); 114.9 (CH, 2); 121.0 (CH, 4); 129.1 (CH, 5); 144.6 (C, 3); 158.8 (C, 1).

### 4.2. HPLC Analysis

High-performance liquid chromatography (HPLC) was carried out using a Vydac 218TP C18 column (250 × 4.6 mm, 5 µm; code: 5103994, Grace, Bannockburn, IL, USA). The chromatographic system was a Shimadzu HPLC-DAD platform (Kyoto, Japan) comprising the following modules: a degassing unit (DGU-20A5R), a solvent delivery unit (LC-20AT), a photodiode array detector (SPD-M20A), an autosampler (SIL-10A HT), a column oven (CTO-20A), and a communication bus module (CBM-20A).

Reversed-phase high-performance liquid chromatography (HPLC) was performed using a binary mobile phase consisting of water and acetonitrile (MeCN), both containing 0.1% trifluoroacetic acid (TFA), at a constant flow rate of 1.0 mL/min. The elution program began with 40% MeCN, held for 1 min, followed by a linear gradient to 85% MeCN over the next 10 min (1–11 min). The gradient then continued to 100% MeCN by 36 min, which was maintained until 42 min. At 42.01 min, the mobile phase composition was rapidly returned to 40% MeCN and maintained until the end of the run at 50 min to allow re-equilibration [54]. Absorbance was monitored using a photodiode array detector over the wavelength range of 190–400 nm. These results are expressed in Supplementary Materials Figures S1 and S2.

### 4.3. In Silico Analysis

In silico analyses of the pharmacological properties of the compounds (solubility, lipophilicity, and pharmacokinetics) were obtained from the results of SwissADME (http://swissadme.ch/index.php#top-21 acessed on 20 september 2022). Properties related to hepatotoxicity, recommended daily dose (mg/kg/day), and mutagenic potential were provided by pkCSM (https://biosig.lab.uq.edu.au/pkcsm/prediction acessed on 20 september 2022). The reference drugs for treating Chagas disease, Benznidazole and Nifurtimox, were included in this stage. Absorption, distribution, metabolism, excretion, and toxicity (ADMET) and Lipinski’s rule of five properties of compounds were assessed as perspectives in an in silico analysis for estimating drug-likeness and oral bioavailability using the Predicting Small-Molecule Pharmacokinetic and Toxicity Properties (pKCSM) approach, according to Neffertiti et al. [55].

### 4.4. Cell Culture

HEPG_2_ cells (ATCC HB8065), used for infection by trypomastigotes and cytotoxicity assays, were cultured in RPMI 1640 medium (Thermo Life Science, Waltham, MA, USA) supplemented with penicillin (100 U/mL, Thermo), streptomycin (130 μg/mL, Thermo), and 10% inactivated fetal bovine serum (FBS, Thermo Life Science, Waltham, MA, USA). The cultures were maintained in an atmosphere of 5% CO_2_ at 37 °C.

The same protocol was used for the HFF-1 cells (ATCC SCRC-1041). However, it was grown in DMEM medium (Dulbecco’s Modified Eagle’s Medium, (Thermo Life Science, Waltham, MA, USA) supplemented with 4 mM L-glutamine (Merck, Rahway, NJ, USA) and 4.5 g/L glucose (Vetec, Speyer, Germany). Epimastigotes (strain Y) were cultured at a temperature of 28 °C, with LIT medium (“Liver infusion tryptose”) supplemented with 10% inactivated FBS (Cultilab, Campinas, Brazil). At 96 h intervals, during the exponential growth phase, 1 × 10^6^ cells/mL were added to a new LIT medium supplemented with SFB. The trypomastigotes and amastigote forms of *T. cruzi* (strain Y) were maintained in epithelial cells of the HFF-1 strain. These cells were grown in culture bottles at 37 °C under a 5% CO_2_ atmosphere in DMEM medium supplemented with 10% fetal bovine serum (FBS, (Thermo Life Science, Waltham, MA, USA). After 24 h incubation, the new cultures were infected with trypomastigote forms of *T. cruzi* (10^7^ trypomastigotes per bottle). These cells were incubated for 2 h, washed with medium, and then reincubated for 120 to 168 h, during which time new trypomastigotes were released into the supernatant [50,51].

### 4.5. Evaluation of the Antiproliferative Activity of the LDT Compounds in the Epimastigote Stage of T. cruzi

The antiproliferative assay using epimastigote forms was performed on a BD Ac-curi™ C6 Plus flow cytometer, with three independent experiments conducted in triplicate for both compounds. 3 × 10^6^ epimastigotes were distributed in 6-well plates in LIT medium supplemented with 10% FBS. Twenty-four hours later, different concentrations of the compounds were added, and the epimastigotes were incubated in an oven at 28 °C for up to 72 h [50]. The concentrations were defined based on previous viability results, specifically: 0.1 μM, 1 μM, 3 μM, 5 μM, and 10 μM. To determine the IC50, control results and those from different treatments were compared using the GraphPad Prism 7 program, as described in the section on statistical analysis.

### 4.6. Evaluation of the Viability of the Trypomastigote of T. cruzi Treated with LDT Compounds

To evaluate the viability of *T. cruzi* trypomastigotes for determining the LD50, trypomastigotes were collected from the culture of infected cells over seven days. The cells were counted in a Neubauer chamber, and 1 × 10^7^ cells were treated with LDT10 or LDT119 at concentrations of 1 μM, 3.0.0 μM, 5.0 μM, and 10 μM for 24 h. Then, the cells were incubated with propidium iodide (PI, Thermo Fisher Scientific) at a concentration of 1 mg/mL for 5 min to facilitate further analysis by flow cytometry [50]. As a positive control, the trypomastigotes were incubated with 0.1% saponin (Sigma Aldrich, St. Louis, MO, USA).

### 4.7. Evaluation of the Proliferation of Amastigotes of T. cruzi Treated with LDT Compounds

HFF-1 cells were infected with trypomastigotes (1:10) for 24 h, and after differentiation to amastigotes, LDT10 and LDT119 compounds were added at concentrations of 0.05 μM, 0.01 μM, 0.5 μM, 1 μM, and 1.5 μM. After 24, 48, and 72 h, the host cells and amastigotes were labeled with Hoescht 33342 (1:5000—Thermo Life Science Waltham, MA, USA), photographed under Leica 6000B optical microscope (Wetzlar, Germany) (100×—N.A = 1.4), and the cell proliferation of intracellular amastigotes was evaluated by nucleus count and results expressed as the number of amastigotes per infected host cells compared to controls incubated without LDT compounds. 600 host cells were counted in each coverslip [50]. The experiments were performed in duplicate in three different trials.

### 4.8. Evaluation of the Cytotoxicity of the Compounds in HFF-1 and HEPG2 Cells

The evaluation was performed using a cell viability test with Resazurin (10 mg/mL), a non-fluorescent blue dye, with absorbance readings taken at 570 nm and 600 nm. The HFF-1 cell line was used to evaluate the cytotoxic potential of LDT10 and LDT119, while the HEPG2 strain was utilized for a specific in vitro assessment of their hepatotoxic potential of these compounds. For the assays, 1 × 10^4^ cells were plated in 96-well transparent plates (TPP), allowed to adhere for 24 h, washed, and then treated with LDT compounds. The concentrations used were 0.5 μM, 1 μM, 3μM, 5 μM, 10 μM, and 20 μM for the compound LDT119 and 0.5 μM, 1 μM, 3 μM, 5 μM, 10 μM for LDT10, for 24, 48, and 72 h. In addition, untreated cells (as a control) and cells fixed with 4% paraformaldehyde (to control for non-viable cells) were plated [50,51]. Each condition of the experiment was performed in triplicate, in three independent trials.

### 4.9. Effects on Reactive Oxygen Species (ROS) Production

After treatment with the LD50 of LDT10 and LDT119 for 4 h, the trypomastigotes (10^6^ cells/mL) were resuspended in PBS pH7.2 and incubated with the cell-permeable probe dichlorofluorescein (H_2_DCFDA) (40 µg/mL) for 30 min at 25 °C. After incubation, the parasites were harvested at 500× *g* for 5 min, resuspended in PBS, and immediately analyzed in a spectrofluorometer using excitation and emission wavelengths of 504 and 529 nm, respectively. Parasites treated with 1 mM were used as positive controls of ROS production [51].

### 4.10. Evaluation of Ultrastructural Changes by Transmission Electron Microscopy (TEM)

The cells (epimastigotes and LLC-MK_2_ cells infected with amastigotes) were washed with PBS, pH 7.2, and then fixed with 2.5% glutaraldehyde in 0.1 M sodium cacodylate buffer (Sigma-Aldrich), pH 7.2, and 4% formaldehyde (EMS), pH 7.4. After fixation, the samples were washed and post-fixed for 40 min in 1% osmium tetroxide (EMS) and 1.25% potassium ferrocyanide. Then, after further washing, the samples were dehydrated in an increasing series of acetone (Merck) concentrations at 30%, 50%, 70%, 90%, and, finally, three times at 100%. Finally, the samples were embedded in epoxy resin (Polybed 812) and cut on an ultramicrotome (Leica UC6), collecting the cuts (50–70 nm) on copper grids of 300 mesh. These sections were contrasted for 40 min with 5% uranyl acetate in distilled water and 5 min with lead citrate [50]. After all processing, the samples were observed under a FEI Spirit transmission electron microscope, operating at 80 KV.

### 4.11. Evaluation of Morphological Changes by Scanning Electron Microscopy (SEM)

Epimastigotes and trypomastigotes treated with the IC50 of the compounds and untreated (control) were washed with PBS (pH 7.2) and fixed with 2.5% glutaraldehyde in 0.1 M sodium cacodylate buffer and 0.5% sucrose. After fixation, the samples were transferred to round coverslips (13 mm) coated with poly-L-lysine (0.1 mg/mL) for 20 min to promote adhesion. Then, they were washed with PBS pH 7.2 and post-fixed for 40 min in 1% osmium tetroxide and 1.25% potassium ferrocyanide under light protection. Then, the samples were dehydrated in an increasing series of ethanol (Merck) at 30%, 50%, 70%, 90%, and, finally, three times at 100%. After dehydration, the samples were dried in the critical point equipment (LEICA EM CPD030), metalized with 5–10 nm platinum (LEICA EM SCD 500), and taken for analysis in the scanning electron microscope (FEI or Jeol) operating between 5 and 10 kV [50].

### 4.12. Statistical Analysis

Statistical analyses were performed using the GraphPad Prism 7 program (GraphPad Software Inc., Boston, MA, USA) and the Analysis of Variance (ANOVA) test. Values of *p* ≤ 0.05 were considered significant. The mean and standard deviation were determined from three independently performed trials.

## 5. Conclusions

The LDT compounds demonstrated significant antiproliferative activity against *T. cruzi*, which showed dose- and time-dependent responses when treated with such com-pounds, and the computational approach presented in this manuscript could be used in the final drug screening stage to confirm that potential inhibitors and suggest that LDT compounds have varied pharmacological characteristics that should be considered in the development of a future (possible) drug.

## Figures and Tables

**Table 1:** Pharmacokinetic properties and in silico toxicity of LDT10, LDT119, BZN, and NFX using the SwissADME and pkCSM programs.

Compound	LDT10	LDT119	BZN	NFX
MW (g/mol)	305.52	415.69	270.33	267.29
LogP	4.77	6.99	0.50	0.54
Solubility	Low	Low	High	High
Lipinski Violation	0	1	0	0
Intestinal absorption	93.01%	89.79%	66%	83.9%
Blood–brain barrier	No	No	No	No
GpP Substrate	No	Yes	Yes	No
GpP Inhibitor	No	Yes	No	No
CYP1A2 Inhibitor	Yes	No	No	No
CYP2C19 Inhibitor	Yes	No	No	No
CYP2D6 Inhibitor	No	No	No	No
CYP3A4 Inhibitor	No	No	No	Yes
Mutagenic potential	No	No	No	No
Hepatotoxicity	No	No	Yes	No
Dose (mg/kg/day)	0.408	0.273	0.254	0.216

## Data Availability

The original contributions presented in this study are included in the article/Supplementary Material. Further inquiries can be directed to the corresponding author.

## Supplementary Materials

The following supporting information can be downloaded at: https://www.mdpi.com/article/10.3390/ph19010030/s1, Figure S1. LDT 10 Retention time = 31.8 min Purity = 98.12%; Figure S2. LDT 119 Retention time = 19.4.8 min Purity = 97.81%.
