# Supplementary material for: Inhibitory Activity of LDT10 and LDT119, New Saturated Cardanols, Against Trypanosoma cruzi
Source: Pharmaceuticals (Basel). 2025 Dec 22;19(1):30. doi: 10.3390/ph19010030 (PMC12844929; doi:10.3390/ph19010030)
Supplement: Supplementary file 1 [file pharmaceuticals-19-00030-s001.zip › pharmaceuticals-3920036-supplementary.pdf]

Supplementary Material

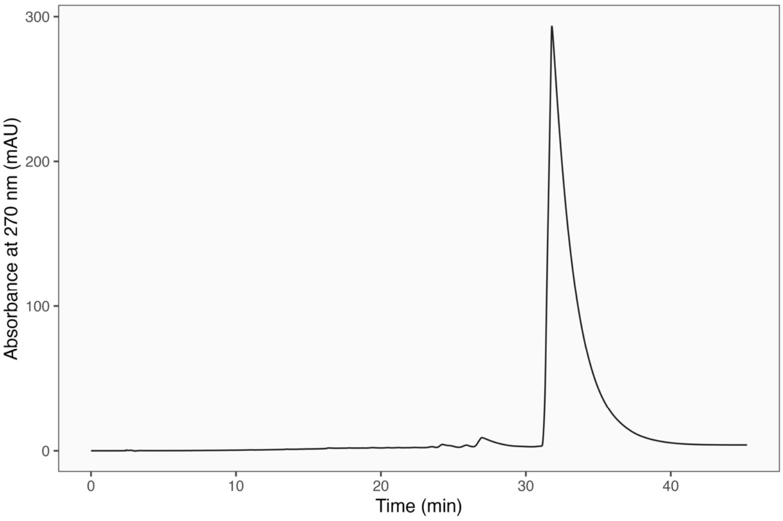

Figure S1. LDT 10 Retention time = 31.8 min Purity = 98.12%.

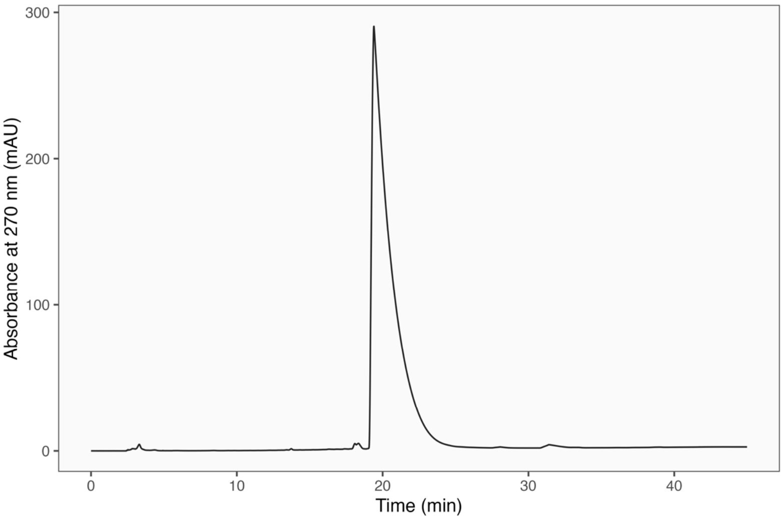

Figure S2. LDT 119 Retention time = 19.4.8 min Purity = 97.81%.
